# Acute cholecystitis: WSES position statement

**DOI:** 10.1186/1749-7922-9-58

**Published:** 2014-11-18

**Authors:** Fabio Cesare Campanile, Michele Pisano, Federico Coccolini, Fausto Catena, Ferdinando Agresta, Luca Ansaloni

**Affiliations:** Division of Surgery, Ospedale San Giovanni Decollato - Andosilla, Civita Castellana, VT Italy; General Surgery Department, Papa Giovanni XXIII hospital, Bergamo, Italy; Department of Emergency Surgery, Maggiore Parma Hospital, Parma, Italy; Department of General Surgery, ULSS19 del Veneto, Adria, RO Italy

## Abstract

**Background:**

The management of acute calculous cholecystitis still offers room for debate in terms of diagnosis, severity scores, treatment options and timing for surgery.

**Material and methods:**

A systematic review about the treatment of acute cholecystitis has been completed. The recommendations of recent guidelines have also been examined taking into account the results of the review.

**Results:**

The evidence available in the literature supports the recommendation about laparoscopic cholecystectomy as treatment of choice for acute cholecystitis. Surgery should be performed as soon as possible after the diagnosis because early treatment reduces total hospital stay and does not increase complication or conversion rates. The antibiotics can play different roles and attention should be posed to the risk of emerging resistance. A surgical or percutaneous drainage of the gallbladder is advocated by some authors in the advanced forms of inflammation or patients with severe co-morbidities; however, the available evidence does not support it, and further studies are necessary to clarify its role.

## Introduction

The literature about the treatment of acute calculous cholecystitis has been recently examined in more than one Consensus Conference [1–3]. The disease could present with a picture ranging from mild, self-limiting, to a potentially life-threatening illness, and the severity of inflammation and its life-threatening potential is also strongly determined by the general condition of the patient. The degree of inflammation of the gallbladder and the conditions of patients with their co-morbidities contribute to indicate the best therapeutic option for every single patient. However, such heterogeneity makes very difficult to standardize a therapeutic protocol for this condition.

The optimal surgical treatment for acute cholecystitis should be examined according to its severity; however, no uniform grading system is yet available, and the need for practical patient-related operative guidelines has been stressed elsewhere [4].

Even the evidences about the outcome of the therapeutic procedures adopted are difficult to evaluate in this heterogeneous context; studies available have not generally examined the optimal treatment for acute cholecystitis according to its severity. A systematic review of the literature must take into consideration this aspect.

The literature review has been presented to the 2nd World Society for Emergency Surgery held in Bergamo in July 2013. This position paper follows the indication emerged in that meeting.

## Review of literature

The results of the systematic literature review performed for the EAES Consensus Conference about the laparoscopic approach to the acute abdomen, published in 2012 were entirely considered for this analysis; the literature search strategy adopted has been detailed in that paper [1].

An additional literature search was done from 2010 through February 2013 with the following limits and filters: adult, clinical trial, review and english language. The PICO (population, intervention, comparison, outcome) system was applied for the MeSH (Medical Subject Headings) search whenever possible. Analogous search has covered the Cochrane Collaboration database and the Google Scholar in order to gather all the remaining evidence, synopses and guidelines on the topic.

The following search string was used:

(“cholecystitis, acute”[MeSH Terms] OR (“cholecystitis”[All Fields] AND “acute”[All Fields]) OR “acute cholecystitis”[All Fields] OR (“acute”[All Fields] AND “cholecystitis”[All Fields]))) AND ((Clinical Trial [ptyp] OR Review [ptyp]) AND (“2010/01/01”[PDAT]: “2013/12/31”[PDAT]) AND “humans”[MeSH Terms] AND English [lang]).

A total of 79 citations was identified.

A search without the limits “clinical trial” and “review” was then carried out, obtaining 392 additional papers whose abstracts were examined for relevance. Two authors (FCC and MP) collected data independently.

After exclusion of duplicates, publications with no abstract and of low interest in the specific topics and key questions, 77 publications were taken into consideration: The papers have been classified for evidence strength following the Oxford CEBM 2011 scheme. In the rest of this paper, the level of evidence obtained has been specified after every relevant reference citation as evidence level (EL) 1–5.

The literature obtained has also been used to update the results of the above cited Consensus Conference. This update has been presented at the 32nd “Congresso Nazionale ACOI” held in Florence (Italy) in May 2013. For the purpose of publication, the search was extended to April 2014.

### Timing of surgery

The issue of early versus delayed laparoscopic cholecystectomy for acute cholecystitis is examined in seven randomized controlled trials [5–11] and 5 meta-analyses (EL1) [12–17]. The studies show that an early treatment reduces total hospital stay and does not increase complication or conversion rates. In particular, the rate of bile duct injury has been shown to be higher in the delayed treated patients, but the difference was not statistically significant due to the small numbers analyzed in the trials.

The optimal delay for surgery after the onset of symptoms it is not completely clarified in the above mentioned studies and deserves a more precise definition. One of the systematic reviews examined above [10, 12] performed a subgroup analysis and could not demonstrate a statistically significant difference between the patients treated less than four days from the onset of symptoms and those of the studies also including patients with a longer delay. One large population-based studies, mentioned above, examined the risk-adjusted association between outcomes and preoperative length of hospital stay (used as a proxy for the onset of symptoms). There was no significant association between preoperative length of stay and 30-day postoperative mortality or overall morbidity. However, patients hospitalized for two or more days preoperatively sustained longer operative times and were significantly more likely to require open cholecystectomy compared with patients who received operation on the day of admission [18]. The above mentioned evidence supported the EAES Consensus Conference to recommend early cholecystectomy for acute cholecystitis, and to state that surgery should be performed as soon as possible after the onset of symptoms.

On the contrary, the authors of the Tokyo guidelines maintain that, despite the demonstration that early cholecystectomy is indicated for acute cholecystitis in an unselected population, still it could be possible to improve the overall outcome of this condition tailoring the treatment according to the severity of the condition and to the patient status. They suggest a staging system based upon severity assessment criteria such as degree of local inflammation and patient conditions. According to their classification acute cholecystitis is defined as “severe (grade III)” if associated with organ dysfunction, “moderate (grade II)” if the completion of a cholecystectomy is likely to be difficult due to local inflammation (“criteria predicting when conditions might be unfavorable for cholecystectomy in the acute phase”), and “mild (grade I)” if it does not meet the criteria for grade II or III [19–21].

Based on that scheme, the Tokyo guidelines suggest early laparoscopic cholecystectomy only in the mild forms of the disease (grade I), in which a laparoscopic cholecystectomy is likely to be easy. In the moderate cases, medical therapy with or without early gallbladder drainage (surgical or percutaneous) followed by delayed cholecystectomy is indicated, except in “experienced” centers. The “severe” grade (with organ dysfunction) is trusted to cholecystostomy.

However, several reports show that early cholecystectomy is safe even in the severe forms of the disease [22–24] or in the elderly population [25–27]. In particular, a recent review of prospective and retrospective series (EL3) [28] did not show an increase in local postoperative complications in severe cholecystitis and confirmed that laparoscopic cholecystectomy is to be considered an acceptable indication for it, despite a demonstrated threefold conversion rate.

### Laparoscopic vs open cholecystectomy

There are two randomized trials (EL2) [29, 30], a population-based outcome research (EL3) [31] and numerous comparative studies demonstrating that laparoscopic cholecystectomy is associated with faster recovery and shorter hospital stay than open cholecystectomy; the population-based outcome research showed also lower morbidity and mortality [31]. This evidence supported the EAES Consensus Conference recommendation that laparoscopic cholecystectomy be the treatment of choice for acute cholecystitis.

The preference for laparoscopic cholecystectomy is also expressed in the Tokyo guidelines [3]; however, the “severity” tailored approach suggested in those guidelines limits the indication for surgery only to the mildest forms of acute cholecystitis and takes in consideration the above evidence only in part (EL5). One of the trials mentioned above, specifically included gangrenous cholecystitis (to be classified in the “moderate” form according to the Tokyo guidelines) [29] and a recent meta-analysis shows that straight laparoscopic cholecystectomy is indicated in severe (gangrenous, empyematous) cholecystitis (EL3) [28].

Advanced age also does not preclude the indication for laparoscopic cholecystectomy (EL3) [32–35].

### Antibiotic treatment

Different antibiotic regimens can be employed in acute cholecystitis; the choice must be based on the most common pathogens, community acquired or health care associated infections, pharmacodynamics and pharmacokinetics of the antibiotic and evolution of sepsis. Moreover, the antibiotics can be administered with different intent: as an ancillary role for early surgery or cholecystostomy or as the unique treatment for acute episode in the non-operative setting and delayed surgery.

The most accredited guidelines arise from TG 2013 and the WSES: the last one is the most updated and take into consideration also “new drugs” such a Tigecycline (see Table 1) [36, 37].

**Table 1:** **Recommendation for antibiotic strategy in acute cholecystitis (modified from WSES 2013)**

Community acquired		Healt care associated	
Infectious situation	***Drug***	Infectious situation	Drug
*No Severe Sepsis ESBL -*	*Amox-Clav*	*No Severe Sepsis*	*Pipera-Tazo + Tigecycline + −Fluconazole*
*No Severe Sepsis ESBL +*	*Tigecycline*		
*Severe Sepsis ESBL -*	*Pipera-Tazo*	*Severe Sepsis*	*Pipera-Tazo + Tigecicline + Echinocandin or Carbap Antibiotics + Teicoplanin + Echinocandin*
*Severe Sepsis ESBL +*	*Pipera-Tazo + Tigecycline + −Fluconazole*		

### Percutaneous cholecystostomy (PC)

Percutaneous tube cholecystostomy (followed or not by surgery) is reported in the literature as an alternative for the emergency treatment in septic high-risk patients. In particular, as mentioned above, the Tokyo guidelines consider the percutaneous (or surgical) drainage as mandatory in the severe grade of acute cholecystitis and also suggest its use in the moderate grade, in order to overcoming the technical difficulties of an inflamed gallbladder. However, gallbladder drainage has never been proven to be an effective alternative to early surgery; the evidence on its role is still poor. The panel of the Tokyo Guidelines states that it is known to be an effective option in critically ill patients, especially in elderly patients and patients with complications; however, no evidence is provided to support the statement, and it is recognized that there have been no randomized controlled trials on the issue.

As a matter of fact, a Cochrane systematic review included two trials, and none of them addressed the comparison of early cholecystectomy vs. percutaneous cholecystostomy [38].

A recent review performed a particularly detailed examination of 53 papers about cholecystostomy as an option in acute cholecystitis (EL3). It found no evidence to support the recommendation of percutaneous drainage rather than straight early emergency cholecystectomy even in critically ill patients. Actually, it suggested that cholecystectomy seems to be a better option for treating acute cholecystitis in the elderly and/or critically ill population [39]. Even if the results obtained from the studies reviewed are non-homogeneous, the mortality rate after PC (15.4%) is significantly higher than reported after early cholecystectomy (4.5%) in published series of similar patients. Of course, the reports analyzed in the PC group take into account also patients who could not have tolerated any surgery, and this limitation has to be considered.

After their review, about 27 further observational studies have been published, confirming that the groups considered in the studies, their inclusion criteria, the results and even the conclusions reached by different authors are largely non-homogeneous. With these limitations in mind, the reported in-hospital mortality for cholecystostomy varies between 4 and 50% (vs. 4.5% reported for cholecystectomy) and its morbidity ranges between 8.2 and 62%.

The role of percutaneous drainage of the gallbladder is difficult to investigate also because different definitions are used to identify “high-risk patients”.

At the present time, percutaneous cholecystostomy cannot be recommended as part of a routine protocol for treatment of acute cholecystitis, but only considered as a possible alternative to reduce anesthesiology risk in a small subset of patients unfit for emergency surgery due to their severe co-morbidities. A randomized controlled trial (CHOCOLATE Trial) has been planned to attempt to clarify the largely conflicting evidence [40].

## Recommendations

We recommend that laparoscopic cholecystectomy be considered the treatment of choice for acute cholecystitis and that surgery be performed as soon as possible after the diagnosis.

The role of antibiotics is relevant both in conservative therapy of the inflammation or as support to invasive procedures. A frequent assessment of the therapeutic protocol is necessary to deal with bacterial resistance. We recommend the use of the 2013 WSES guidelines because they take into account several new drugs compared to TG13.

At present, we do not recommend that cholecystostomy (surgical or percutaneous) be included in routine protocols for treatment of acute cholecystitis, and we suggest that it be considered, only in those patient clearly unfit for emergency surgery, until better evidence are available.

## Discussion

The above reported literature review clarifies the advantages of early laparoscopic cholecystectomy in an unselected population. It has to be stressed, however, that acute cholecystitis is a very heterogeneous disease as far as general and local factors are concerned. The severity of inflammation and its life-threatening potential is strongly determined by the general status of the patient, and the choice of surgical treatment cannot disregard this aspect [4]. Also, the general principles expressed in the literature have to be applied taking into account the environment, the socio-economic context in which such an emergency develops and the related logistics.

The Tokyo guidelines attempted to address such heterogeneity with the above described classification, that takes into account both local and general factors, and a therapeutic protocol based on such scheme.

Such a tailored approach, however, has not been validated by studies showing an improved outcome after its introduction, and a retrospective series did not find any significant benefit [41]. It ends up in a large use of delayed cholecystectomy, despite several meta-analysis of RCTs establish that early laparoscopic cholecystectomy is to be considered the gold standard. It also disregards the examined literature about the safety of early laparoscopic cholecystectomy in the severe forms of the disease. Furthermore, the role of percutaneous cholecystostomy is far from being established as discussed above: its therapeutic potential needs to be confirmed by the literature evidence and integrated in an evidence-based algorithm.

The risk of early surgery for acute cholecystitis should be evaluated against a well-established risk score in order to identifying the patients with reduced functional reserve who should undergo a treatment alternative to surgery. However, none of the available clinical scores for the evaluation of surgical risk for acute conditions [42, 43] has been validated for this disease. Further investigations could help in validating a clinical score useful for clinical and therapeutic decision making for acute cholecystitis.

In addition, it has to be stressed that the general principles expressed in the evidence based recommendations have to be adapted to the technical, environmental and social conditions of the different areas of the world. The indications suggested by the Tokyo Guidelines, too limited for most of the Western countries, still have been shown to increase the adoption of early laparoscopic cholecystectomy in a Japanese context, as reported by Asai [44].

In many developing areas complex technology is not readily available, the diffusion of laparoscopy is still extremely sparse [45] and percutaneous drainage is not available. It is still debated if the introduction of minimally invasive surgery is proper in many developing countries where safe performance cannot always be assured [46] and economic resources could be better employed on more essential health programs [47]. The interpretation of our recommendations in those environments has to take into account that the value of early cholecystectomy for acute cholecystitis is supported by a level of evidence stronger than the laparoscopic approach. Therefore, in those areas where laparoscopy is not readily available, an early laparotomic operation may be preferred to a long transfer to a far away facility, taking into consideration the local situation, the logistics and the patient conditions.
